# Magic mirror on the wall: Cross-buying at the point of sale

**DOI:** 10.1007/s10660-023-09687-4

**Published:** 2023-04-05

**Authors:** Carsten D. Schultz, Björn Gorlas

**Affiliations:** 1 Faculty of Business Administration and Economics, University of Hagen, Universitätsstraße 11, 58097 Hagen, Germany; 2 FOM University of Applied Sciences, Essen, Germany

**Keywords:** Augmented Reality, Cross-Buying, Magic Mirror, Point of Sale, Retailing

## Abstract

Stationary retailers may introduce new technologies, such as augmented reality, to provide product information and recommendations and thus improve shopping experience. Examples of such augmented reality applications are magic mirrors that enable virtual try-on and may induce cross-buying intention. Based on an experimental scenario and the corresponding results from 301 questionnaires, we find that magic mirrors positively impact consumers’ cross-buying intention. Cross-buying behavior depends particularly on price attractiveness and the aesthetic appeal of the products. Further, men place less emphasis on price attractiveness when considering cross-buying options than women. Whereas magic mirrors reduce the relationship between perceived product benefits and cross-buying behavior, the technology increases the positive effect of perceived convenience on consumers’ buying behavior. Magic mirrors, thus, improve convenience but hinder the perception of product benefits – these are better communicated by sales assistants. Magic mirrors may only partly replace sales staff, but retailers can combine both to serve consumers better.

**Supplementary Information:**

The online version contains supplementary material available at 10.1007/s10660-023-09687-4.

## Introduction

Digitalization is a dominant and ongoing trend affecting all areas of life. Digital technologies and developments such as mobile and wearable devices, crowdsourcing, cloud solutions, internet of things, virtual and augmented reality, and artificial intelligence transform everyday life [35]. Digitalization affects, for example, both organizational buying [7] and selling behavior [62], creates business processes, such as crowdfunding [47], dynamic pricing [90], or open innovation [55], and is reshaping the retail industry [27]. Changes in retailing are directly perceivable by consumers, such as the introduction of online shops into a network of physical stores [29], or the integration of digital devices and services [22]. The COVID-19 pandemic further accelerated the digitalization of retailing that has received more and more research attention [16], [61]. Augmented reality is an example of digitized services in retailing that enables consumers to interact with and visualize products [38], [64].

Augmented reality – considered as a three-dimensional reproduction of virtual objects in a real environment [3] – thus enhances consumers’ retail shopping experience and reduces the need for haptic contacts, supporting social distancing efforts. An in-store augmented reality application is the so-called magic mirror. Magic mirrors allow consumers to virtually try on apparel [33] and accessories [79]. Beyond singular product presentation, magic mirrors can also provide consumers with cross-selling offers that match their originally chosen product [79]. The displays of magic mirrors can also provide additional information to reduce the potential information deficit in comparison to online shopping. Augmented reality applications may thus provide support for stationary retailing when competing with online retailers and marketplaces.

Cross-selling describes the strategical measure of retailers, whereas cross-buying refers to the corresponding consumer behavior. Given the competitive pressure of online retailing, cross-buying behavior is increasingly important for stationary retailers and has numerous advantages from a company perspective [57]. For example, retailers can lower customer churn, increase consumer loyalty, and obtain higher consumer lifetime valuation [2], [45]. Overall, cross-selling means and corresponding cross-buying behavior need less investment in the sales process than in acquiring new customers. Cross-selling can, thus, increase revenue and profit contribution per consumer as well as retain consumers while also increasing customer loyalty. Cross-buying behavior also allows for complex pricing and assortment strategies [42]. Traditional approaches to increase cross-buying at the point of sale, such as sales staff and product placement [34], are limited. The use of augmented reality, especially magic mirrors, can potentially overcome the traditional limitations in cross-selling but has received limited attention in research on stationary retailing and cross-buying behavior. As cross-buying highly depends on a retailer’s marketing effort [45], augmented reality provides a valuable measure to leverage their brand by maximizing consumer revenues and profit contributions.

Cross-selling via magic mirrors further contributes to the understanding of augmented reality marketing in branding, inspiring, convincing, and keeping customers [71]. In particular, cross-selling via magic mirrors can add to all four dimensions of the so-called BICK framework. For branding purposes, the cross-selling offer builds brand awareness for the additional product, presents such product offerings, and thereby helps to reach more customers. Magic mirrors also strengthen the brand image of the retailer. Such services also inspire customers and trigger customer needs. Most particular, cross-selling can convince customers and thus generate sales and increase profit contribution [45]. Lastly, if customers perceive benficial service through cross-selling offers via magic mirrors, this augmented reality instrument fosters customer experience and increases customer loyalty – overall, contributing to keeping customers.

The present study adds to the existing literature by examining the impact of augmented reality on consumers’ cross-buying intention at the point of sale. This study examines differences in cross-buying intention between a shopping situation with augmented reality and a shopping situation without augmented reality. In an online-scenario survey, participants evaluate the attractiveness of a cross-selling offer and reveal their cross-buying intention. Using a multiple-dimension approach [52], the perceived product benefits, price attractiveness, convenience, and fit form the attractiveness of the cross-selling offer. The study thus contributes in multiple ways to research and practice. This study investigates the potential impact of augmented reality on the attractiveness of the cross-selling offer and subsequently consumers’ cross-buying intentions. Firstly, the empirical results highlight the importance of perceived price attractiveness and product aesthetic quality. Secondly, magic mirrors show a two-sided effect, such that they decrease the perceived product benefit effect and increase the perceived convenience effect on consumers’ cross-buying behavior. Magic mirrors may, thus, replace sales staff only at the cost of perceived product benefits but enable retailers to attend to more customers and create cross-selling offers for each. Magic mirrors may also positively affect perceived product aesthetics. Lastly, the study reveals that women perceive price attractiveness as more relevant than men.

The remainder of the paper is organized as follows. The next section provides a brief background on cross-buying behavior and augmented reality. The methodology section then outlines the research design and presents the data collection and sample. Afterwards, we analyze the data and discuss the empirical results. The paper concludes with some implications, limitations, and future research.

## Background

### Cross-buying behavior

Consumer relationship management generally follows three distinct phases: consumer acquisition, consumer loyalty and retention, as well as consumer recovery. In the second phase of the customer-company relationship, consumers increase their purchasing behavior and enter into a closer relationship with the company. This phase predominantly considers cross-buying which is, thus, a relevant determinant of customer value [46]. Following Kumar et al. [45], this study defines “cross-buy as the total number of different product categories that a consumer has purchased from a firm from the time of the first purchase.” Literature investigates several factors influencing cross-buying behavior and intention (e.g., [45], [57], [88]). Existing research has, for example, studied consumer satisfaction [3], commitment of consumers towards a company [88], price and price-performance ratio of products [10], fit between products [57], prepayment options [39], and shopping convenience [50] as antecedences of cross-buying.

Fostering cross-buying behavior is, thus, central to consumer retention [46]. An increase in purchases from the same company can extend consumers’ relationship with the company [72] and increase purchase frequency [73]. Beyond the increase in revenue, cross-buying behavior leads to higher consumer engagement, increased profit contribution, and higher switching costs [45]. However, companies need to be careful to identify profitable consumers because cross-buying may not always result in higher consumer profitability [77].

Maitzen [52] summarizes four categories of antecedences of cross-buying behavior: relationship-related factors, provider-related factors, consumer-related factors, and performance-related factors. Relationship-related factors of cross-buying behavior are consumer satisfaction [48], trust [81], commitment [88], loyalty [87], and the length of the business relationship [74]. In the case of performance-related factors, previous research has studied, for example, price and price-performance ratio [14], [89], as well as the fit between products (complementary products) [21], and the fit between additional products and the retailer [52]. Provider-related factors include research on marketing activities [45], company image [51], and the role of the sales staff. The consumer-related factors include convenience in the buying process (e.g., one-stop-shopping) [57] and consumer sociodemographics, such as age [45]. Previous research has considered traditional instruments for promoting cross-buying, such as coupons [5], [19] or sales staff [34]. However, research still lacks understanding of the effect of augmented reality on cross-buying.

### Augmented reality in marketing

Augmented reality is part of the reality-virtuality continuum [53]. Rauschnabel et al. [70] provide a new lens in the xReality framework by anchoring augmented reality in local presence. The augmented reality-enriched environment adds virtual objects to reality [4]. Augmented reality is primarily used in online shopping for product presentation and visualization [40]. In stationary retailing, only a few companies have been testing the potential of augmented reality. For example, augmented reality mirrors present cosmetics or glasses on a display with front cameras [30], [70]. Fashion stores have similarly used so-called magic mirrors to enable virtual try-on of clothes by consumers [40], [59].

Research on augmented reality has primarily concentrated on its technical feasibility but now moved on to its application, for example in the marketing domain. In general, research on augmented reality in marketing is scarce so far. Kumar [43] provides a recent systematic review and points out the need for augmented reality research and experiential value. Rauschnabel et al. [69] define augmented reality marketing as “a strategic concept that integrates digital information or objects into the subject’s perception of the physical world, often in combination with other media, to expose, articulate, or demonstrate consumer benefits to achieve organizational goals.” In a recent proposition to capture a broad, goal-oriented, and interdisciplinary understanding, augmented reality marketing is defined “as the strategic integration of AR experiences, alone or in combination with other media or brand-related cues, to achieve overarching marketing goals by creating value for the brand, its stakeholders, and societies at large, while considering ethical implications.” [71, p. 1141].

Previous studies on augmented reality focus mostly on attitudes, motivations, or reactions to the application of augmented reality, in particular the devices [69]. An initial theme was consumer acceptance of augmented reality [75]. Huang and Liao [32], for example, integrate consumers’ desire for innovations into consumer acceptance of augmented reality. The authors summarize that consumers with high innovativeness put more emphasis on usefulness, aesthetics, and service excellence, whereas consumers with low innovativeness value playfulness and ease of use of the augmented reality application. An organizational adoption perspective reveals that firms’ technology competence, relative advantage, and top management support, as well as consumer readiness determine the organizational adoption of augmented reality for e-commerce [17].

A recent overview integrates augmented reality in four common objectives: branding, inspiring, convincing, and keeping [71]. The so-called BICK framework for augmented reality marketing proposes a processual perspective to capture various phases within the customer journey. Branding refers to building brand awareness, product knowledge, and brand image, whereas inspiration, for example, triggers needs and wants. Aspects of convincing summarize phases that generate interest to purchase and enforces willingness to pay. Lastly, keeping aggregates aspects of customer retention, such as after services, added value services, and customer loyalty.

Augmented reality also provides opportunities for retailing, including in-store, online, and mobile-based applications [15]. Willems et al. [92] offers a comprehensive inventory of such retail technologies and summarize that the majority provides cost savings, convenience, and utilitarian benefits, whereas few approaches offer hedonic or symbolic benefits. Retailing augmented reality technologies may rely on markers, such as barcodes, QR codes, and RFID tags, or operate markerless, for example using image recognition [75]. Compared to traditional shopping experiences, consumers perceive the following six shopping benefits through augmented reality: (a) more product information, (b) buying decision support, (c) greater product choice and variety, (d) virtually trying out products, (e) product demonstrations, and (f) product personalization [18]. Examples of in-store augmented reality applications are augmented labels, smart displays, magic mirrors, and virtual fitting rooms [15]. Such applications generally use projection-based interfaces that offer consumers an enhanced, immersive, and interactive shopping experience. The cross-buying function addressed in the present study is particularly relevant for magic mirrors and their use in virtual fitting rooms. Product information, consumers’ imagery, and the sense of psychological ownership positively affect their product evaluation when using such augmented reality applications [40]. Consumers’ perceived usefulness, entertainment, and value predominantly drive their attitudes towards using magic mirrors [56]. Further, magic mirrors can potentially enhance service quality and customer satisfaction [59].

Along the factors that influence cross-buying behavior, we briefly consider the state of research addressing augmented reality. These four factor groups are relationship-related, provider-related, consumer-related, and performance-related [52]. Some relationship-related factors have been examined and confirmed, for example, that augmented reality may increase consumer satisfaction [68]. When using augmented reality, experience increases consumer satisfaction [13]. For example, a qualitative study supports this relationship in stationary retailing [59]. Augmented reality creates a positive shopping atmosphere [67]. Regarding mobile augmented reality applications in shopping centers, consumers associate these applications with cognitive (e.g., knowledge, awareness) as well as emotional (e.g., enjoyable and stimulating experiences) benefits [58]. Provider-related factors are rarely considered in the cross-buying literature. An exception is the use of augmented reality as exclusive advertising by Woods [13]. Another aspect is perceived convenience as a consumer-related factor. In general, augmented reality is found to have a positive impact on convenience [14], [68]. Augmented reality helps reduce search and transaction costs and increases convenience in consumer decision-making [30]. There is evidence supporting this notion in stationary retailing [33], [92]. Such digital recommendations can, thus, improve the perceived purchase decision quality by consumers. Research on augmented reality has not regarded performance-related factors so far. For example, the impact of augmented reality on the price perception of consumers has not been studied. However, previous research suggests that product tests are more effective than advertising as consumers can actually evaluate the product [80]. Augmented reality also positively influences utilitarian and hedonic customer value perceptions [30]. A recent meta-analysis confirms utilitarian and hedonic benefits for users’ intention to use augmented reality [44]. In consequence, we assume virtual product tests through augmented reality to have a positive impact on price perception. Augmented reality applications that include recommendation functionalities may also have the potential of quickly comparing alternatives [66], [80]. In addition, Hilken et al. [30] indicate that augmented reality positively affects word-of-mouth behavior.

### Research model and hypotheses

#### Research model

**Fig. 1:**
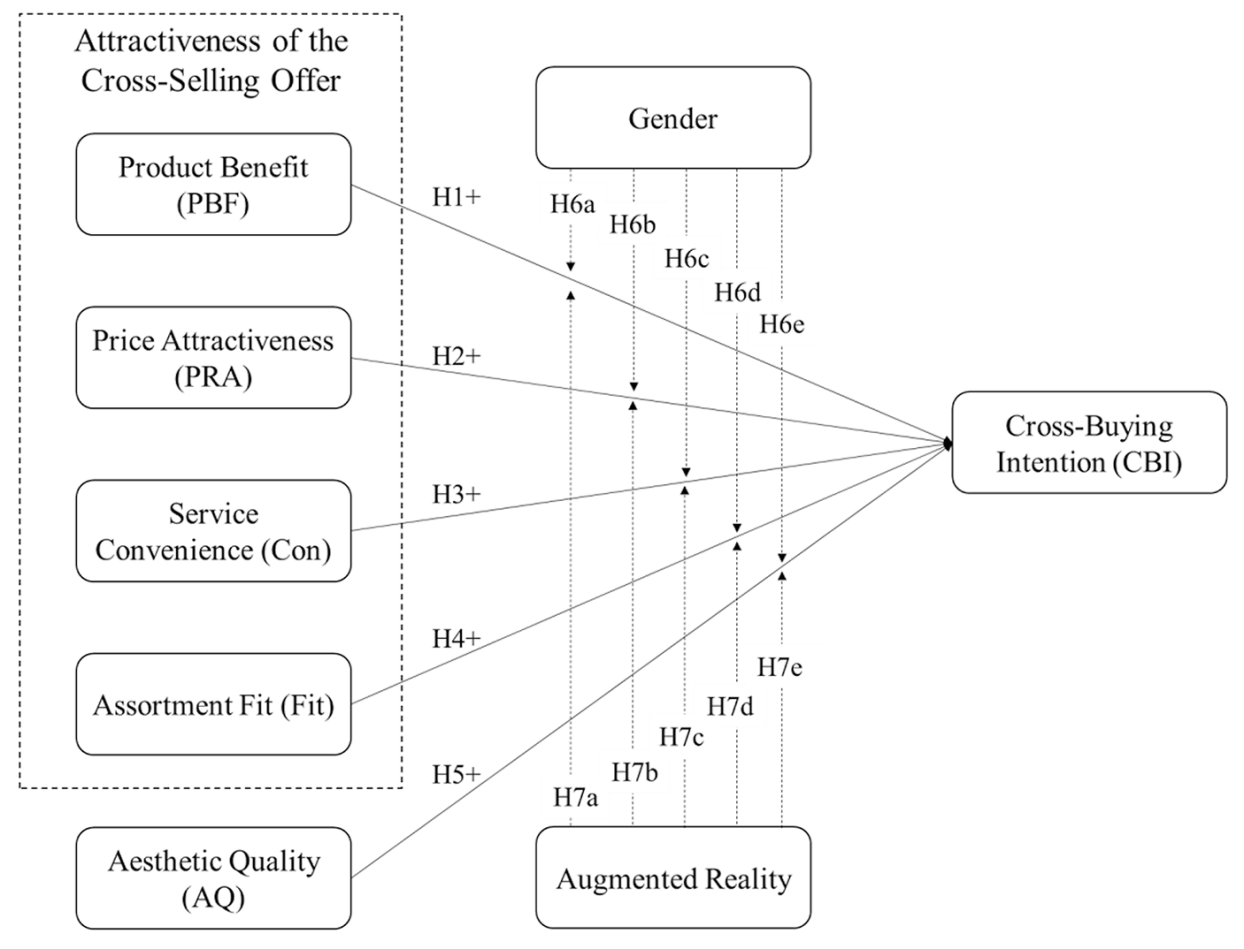
Research Model

The attractiveness of the cross-selling offer directly affects the cross-buying intention. The attractiveness of the cross-selling offer has been defined as the evaluation of an offer by existing or potential consumers based on their perception of the product’s attractive and beneficial effects [52]. This study draws on Maitzen’s categorization [52] and utilizes four constructs to measure the attractiveness of the cross-selling offer. These constructs are (a) product benefit, (b) price attractiveness, (c) convenience, and (d) fit. Maitzen introduced product benefit to generalize findings from the predominantly researched financial industry, while the other three constructs are based on previous literature. Perceived benefit describes the extent to which potential buyers perceive the cross-selling offer as fundamentally useful and beneficial. As such, product benefit refers to the product utility and its uses. Perceived price attractiveness then describes the extent to which prospects perceive the cross-selling offer as having a good price-performance ratio. Convenience refers to the extent of the procurement effort. As such, it represents the extent to which prospects perceive a reduction in the buying effort through the cross-selling measure. Lastly, the perceived fit presents the degree of congruence between the original product and the cross-selling offer. This study introduces augmented reality as a moderator in these relationships. We also control for gender and include the perceived aesthetic quality of the products in the research model. Figure 1 displays the resulting research model.

#### Attractiveness of the cross-selling offer

Previous research indicates that interactions of consumers with an offer create affective responses, such as pleasure, and enhance the ability of consumers to evaluate the offer [26]. Such improved evaluation reduces the purchasing risk and leads to an increased willingness to buy. Accordingly, Abrar [1] found a positive impact of augmented reality on purchase intention in online shopping. Following this line of argumentation, the present study assumes a positive effect of augmented reality on cross-buying intention. The cross-buying intention is itself subject to the attractiveness of the cross-selling offer. Following Maitzen, (a) product benefit, (b) price attractiveness, (c) convenience, and (d) fit determine the attractiveness of the cross-selling offer [52]. The four dimensions are based on social exchange theory (e.g., [31], [86]) and schema theory (e.g., [6]).

Following the understanding of social exchange as the exchange of rewarding or costing (tangible or intangible) activities between at least two people/groups [31], a positive difference between reward and cost is a prerequisite for maintaining and intensifying future interactions. Maitzen concludes that product benefit, price attractiveness, and convenient availability of alternatives construe the attractiveness of a cross-selling offer in a specific situation.

A general foundation of various conceptualizations of schemata is the assumption that individuals classify, store, and retrieve information in memory structures (e.g., [6]). In consequence, the fit of the cross-selling offer increases its overall perception and, thus, positively contributes to the attractiveness of the cross-selling offer [52].

Following this conceptualization, product benefit, price attractiveness, convenience, and fit create a positively perceived cross-selling offer which, in turn, positively influences consumers’ cross-buying intention (H1-H4).

##### H1

Product benefit of the cross-selling offer positively affects the cross-buying intention.

##### H2

Price attractiveness of the cross-selling offer positively affects the cross-buying intention.

##### H3

Service convenience of the cross-selling process positively affects the cross-buying intention.

##### H4

Assortment fit of the cross-selling offer positively affects the cross-buying intention.

#### Aesthetic quality

The study also considers the product aesthetics on the cross-buying intention. Aesthetics originate in the fine arts domain, where they are viewed in terms of stimulus-related beauty and appeal [8]. Aesthetic factors, for example, influence quality perception, attractiveness, and download intention of mobile apps [8]. Visual aesthetics can also enhance pleasure and satisfaction and predetermine a pleasurable consumer experience [54]. Product aesthetics combined with utilitarian benefits create important first impressions and long-term consumer satisfaction in the fashion industry [9]. In the case of cosmetics augmented reality, Tan et al. [85] support that breadth of product appeal is positively associated with product sales, whereas the sales impact of augmented reality is stronger for brands with a narrower appeal. Overall, previous results indicate a positive effect of aesthetic quality on the cross-buying intention.

##### H5

The perceived aesthetic quality of the products positively affects the cross-buying intention.

#### Gender in augmented reality

Stereotypically, ‘men buy, women shop’ [91]. As such, women embrace the shopping experience whereas men prefer to efficiently complete the shopping process [60]. Correspondingly, men are more concerned with wait expectations and value store atmospheres less than women [24]. Women are also willing to pay more for the same product when it is offered in a hedonic store atmosphere [11] and have generally higher expenditures on fashion purchases [63]. Gender also shows significant differences in online shopping, in that men have more favorable attitudes to online shopping than women, whereas social influences and privacy concerns are more pronounced for women [36]. Results on potentially different gender perceptions of augmented reality have been scarce. Kheiravar and Richter [37] indicate that men are more likely to accept using magic mirrors than women. Similarly, the psychological perspective of one’s body image may negatively affect women’s use intention of virtual try-on [94].

##### H6a-H6d

The positive effect of the attractiveness of the cross-selling offer on the cross-buying intention is moderated by consumers’ gender.

Similarly, gender can impact the perception of the aesthetic product quality and its impact on cross-selling behavior. Thus, the model controls for this moderation effect.

##### H6e

The positive effect of the aesthetic quality on the cross-buying intention is moderated by consumers’ gender.

#### Magic mirror and cross-buying

Following the general line of argument above, the study assumes that the use of the magic mirror moderates the positive effect of the attractiveness of the cross-selling offer on the cross-buying intention. Consequently, we next present the four corresponding moderating hypotheses across the four dimensions of the attractiveness of the cross-selling offer.

Augmented reality applications may communicate consumer benefits [69]. Realizing its benefits can lead to a higher valuation of a product. Even if perceived as marketing communication, some research suggests potential upsides if the communication is not excessive [41], [78]. Additionally, consumers positively perceive the provided product recommendations that fit the original products. In particular, if additional information is well aligned, more information leads to higher quality perception and higher price acceptance [66]. Thus, recommendations via an augmented reality application has a positive impact on perceived product benefits.

##### H7a

The positive effect of perceived product benefit on cross-buying intention is positively moderated by the use of augmented reality.

As mentioned above, augmented reality positively affects the perceived atmosphere in the store [67]. Augmented reality creates positive emotions and, thus, increases behavioral intentions and shopping value. Similarly, research on recommender systems suggests that providing more information leads to perceived higher quality and, thus, to higher price acceptance [66]. Following an eMarketer survey, 93.3 million (28.1% of the US population) are expected to use augmented reality at least once per month in 2021 – forecasting to rise to 110.1 million in 2023 [65]. This follows the perception of product trials creating service value [80]. In turn, such augmented reality service creates a positive price perception and, in consequence, positively affects the attractiveness of the cross-selling offer.

##### H7b

The positive effect of perceived price attractiveness on cross-buying intention is positively moderated by the use of augmented reality.

At the point of sale, digital technologies influence convenience via customization and emotional shopping experience [49]. Consequently, consumers also rate information services, such as augmented reality applications, positively at the point of sale [92]. In a setting of makeup, magic mirrors as one augmented reality application particularly increase shopping convenience [33]. The virtual makeup application saves time and reduces transaction and information costs. The present study correspondingly assumes a positive influence of augmented reality on convenience.

##### H7c

The positive effect of perceived convenience on cross-buying intention is positively moderated by the use of augmented reality.

One challenge in cross-buying situations is the complexity of imagining how a product fits a consumer in combination with another product [30]. Applying the cross-selling offer to the consumer through an augmented reality application relieves complexity and consumers’ mental load. Such effects are shown for furniture placement and fitting of sunglasses [30]; a comparable reduction in complexity is reasonable for apparel (stationary) retailing. By simplifying the evaluation for consumers, consumers may better evaluate the product and cross-product fit, which, in turn, leads to higher attractiveness of the cross-selling offer.

##### H7d

The positive effect of perceived fit on cross-buying intention is positively moderated by the use of augmented reality.

Lastly, the use of magic mirrors may also influence the perceived aesthetic quality of the products. As the aesthetic quality may, in turn, influence the cross-buying behavior [9], [85], we thus propose a moderating effect of augmented reality on the relationship between aesthetic quality and cross-buying behavior.

##### H7e

The positive effect of perceived aesthetic quality on cross-buying intention is positively moderated by the use of augmented reality.

## Methodology and empirical analysis

### Experimental setup

**Fig. 2:**
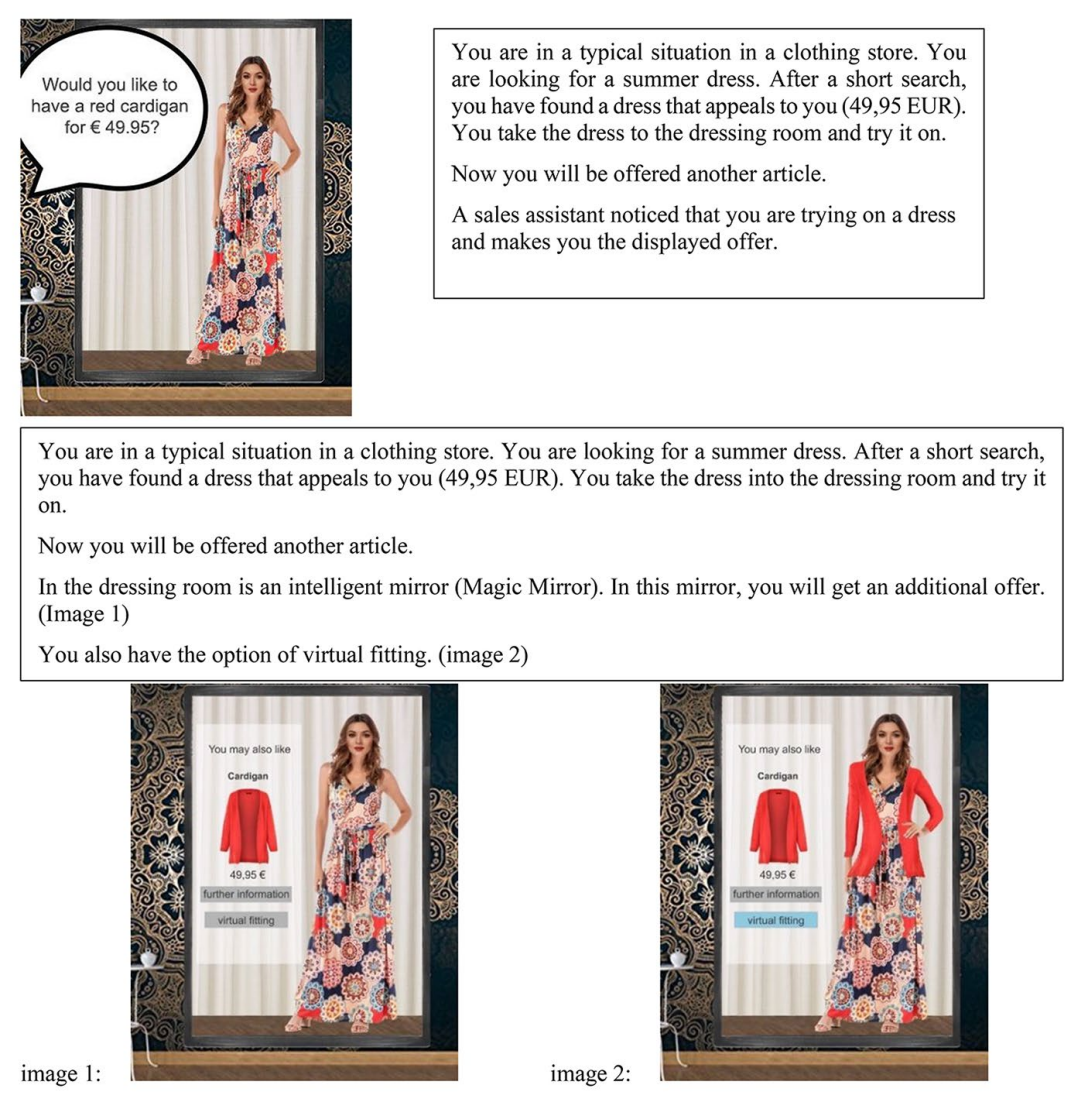
Scenario for Women without and with Augmented Reality

The present study employed an experimental scenario technique and collected data via an online survey. To control for external factors (e.g., behavior of employees), an online experiment was carried out. The participants were, thus, presented with different scenarios by means of text and images before answering the questionnaire. The procedure followed established routes in consumer behavior and augmented reality [57], [77]. First, the survey asked for sociodemographics and technology use. After that, the interviewee was randomly shown one of two scenarios based on the participants’ gender. One scenario included a cross-selling offer without augmented reality. The alternative scenario included a magic mirror as an augmented reality technology to present a cross-selling offer. This is similar to the scenario in Sjøbakk et al. [79]. The scenarios were also created gender-specific, resulting in four scenarios (2 with/without augmented reality x 2 female/male). Figure 2 presents the scenario for women.

### Measurement

The present study used multi-item measurement for data measurement. Product benefit, price attractiveness, convenience, fit, and cross-buying intention are based on existing literature. For product benefit, items were drawn from [82]. Price attractiveness was assessed with items from [25] and [83]. Items for convenience were adapted from [76]. Items based on [52] were used for the fit. The cross-buying intention was addressed by items from [20] and [84]. All items were measured on a 7-point rating scale. We also registered the aesthetic quality of products with three items.

### Data sample and data overview

A pretest suggested minor changes in question phrasing. The online survey was then carried out in April 2019. In order to ensure data quality, we collected participants’ consent for the study at the beginning of the survey and directly asked about the seriousness of their participation at the end of the survey. Furthermore, the survey used items from established scales, and the items were randomly rotated in each questionnaire. We also controlled for straightlining behavior but found none in the final sample.

The questionnaire was opened 462 times and yielded a total of 317 completed questionnaires. All participants consented to anonymously and voluntarily participate in the study being able to terminate the study at any time without negative consequences. We removed two questionnaires based on a seriousness check and 14 minors from the sample, resulting in a final sample of 301. Participants were 52.5% (158) female and 47.5% (143) male. Following the random assignment of participants to the augmented reality and non-augmented reality scenarios, gender was also almost equally distributed in the case of the magic mirror (female 53.0% (80); male 47.0% (71)), and the sales assistant scenario without the magic mirror (female 52.0% (78); male 48.0% (72)). The majority of our participants was aged between 20 and 40 (73.1%) and resided in Germany (93.7%). We also asked for education and occupation but found no differences across these demographics. The scenarios with augmented reality and without augmented reality were randomly assigned and no significant demographic differences were detected.

We conducted power analysis in G*Power to determine the minimum required sample size. To explore the modeled associations at a 0.05 significance level, an effect size of 0.15, and a power level of 0.80, the suggested minimum sample size is n = 146 to perform the variance-based structural modeling analysis. The final sample of 301 questionnaires, thus, exceeds the minimum sample size of n = 146.

### Empirical results

We conducted variance-based structural equation modeling to analyze the associations depicted in the research model (Fig. 1). Calculations are done with SmartPLS 4. For significance tests, we run bootstrapping with 5,000 subsamples. We first control all measurement models before assessing the quality of the structural model. Afterwards, we present the results for the hypotheses.

The measurement models are reflective for all latent constructs. To assess the multi-item measurement, we inspect the individual item reliability, construct reliability, and discriminant validity. The criteria are a factor loading of ≥ 0.7 at a 5% significance level (Table 1), establishing item reliability. Average variance extracted (AVE) ≥ 0.5, Cronbach’s Alpha ≥ 0.7, and Jöreskog’s Rho ≥ 0.7 provide support for construct reliability (Table 2). Finally, the heterotrait-monotrait (HTMT) criterion ≤ 0.85 [28] confirms discriminant validity (Table 3). All indicators exceed the relevant threshold levels (see Tables 1 and 2, and 3).

**Table 1:** Overview of the measurement items and item reliability

Variable	Item	Statement	Loading
Product Benefit			
	PBF1	The additional service (cardigan) is helpful.	0.898
	PBF2	The additional service (cardigan) seems to be useful in many situations.	0.940
	PBF3	I recognize the general usefulness of the additional service (cardigan).	0.860
	PBF4	The additional service (cardigan) appears to be beneficial.	0.930
Price Attractiveness			
	PRA1	The additional offer (cardigan)is reasonably priced.	0.911
	PRA2	The additional service (cardigan) offers value for money.	0.914
	PRA3	If I had purchased the additional offer (cardigan) at this price, I feel like I was getting my money’s worth.	0.907
	PRA4	I feel like I’m getting the additional offer (cardigan) at a reasonable price.	0.939
Convenience			
	CNV1	It is convenient to accept the additional offer (cardigan) since I am already in the store.	0.884
	CNV2	The purchase of the additional offer (cardigan) is easier for me than visiting different retailers.	0.921
	CNV3	The additional offer (cardigan) saves me time searching for alternatives.	0.911
	CNV4	Before I buy, I can easily determine if the additional offer (cardigan) is what I need.	0.772
Fit			
	FIT1	The additional offer (cardigan) fits the range of the retailer.	0.811
	FIT2	I wear the original product (summer dress) and the additional offer (cardigan) in similar situations.	0.846
	FIT3	The price of the additional offer (cardigan) fits the prices of the retailer.	0.777
	FIT4	Combined use of the original (summer dress) and additional offer (cardigan) is favorable.	0.898
Cross-Buying Intention			
	CBI1	If I were to accept an offer, it would probably be this offer (cardigan).	0.898
	CBI2	There is a high probability that I will accept this offer (cardigan).	0.952
	CBI3	I am definitely considering this offer (cardigan).	0.942
	CBI4	I am willing to accept this offer (cardigan).	0.935
Aesthetic Quality			
	AQ1	I like the original product (summer dress | polo shirt).	0.742
	AQ2	I like the additional product offer (cardigan).	0.878
	AQ3	I like the combination of the product and the additional offer	0.913

Note: All loadings are significant at the 0.001 level.

**Table 2:** Construct reliability and convergence validity

Latent Variable	Cronbach’s α	Dillon-Goldstein’s ρ	AVE
Product Benefit	0.928	0.929	0.824
Price Attractiveness	0.938	0.940	0.844
Convenience	0.896	0.904	0.764
Fit	0.855	0.866	0.698
Aesthetic Quality	0.802	0.827	0.719
Cross-Buying Intention	0.949	0.951	0.868

**Table 3:** Heterotrait-monotrait results (HTMT)

Variable	PBF	PRA	CNV	FIT	AQ	GD	AR	CBI
Product Benefit (PBF)								
Price Attractiveness (PRA)	0.541							
Convenience (CNV)	0.725	0.641						
Fit (FIT)	0.735	0.619	0.793					
Aesthetic Quality (AQ)	0.376	0.457	0.330	0.297				
Gender (GD)	0.107	0.250	0.059	0.124	0.192			
Augmented Reality (AR)	0.457	0.294	0.466	0.433	0.080	0.010		
Cross-Buying Intention (CBI)	0.628	0.735	0.786	0.663	0.542	0.066	0.421	

The inner structural model is examined based on the path coefficients > 0.1, significance of the path coefficients at a 5% level, coefficient of determination R² ≥ 0.1, predictive relevance Stone-Geisser Q² > 0, and effect sizes f² ≥ 0.02. R² is 0.720 for cross-buying intention. Thus, this model accounts for 72.0% of the variance in cross-buying intention. Q² is 0.538, hence, it establishes predictive relevance. Effect sizes f² are small for every significant relationship following Cohen’s categorization.

The four dimensions of attractiveness of the cross-selling offer establish one significant main effect. Price attractiveness positively influences consumers’ cross-buying behavior (H2: β = 0.368, *p* < 0.001), confirming H2. The relationship between fit and cross-buying behavior is positive but just above the 5% level (H4: β = 0.146, *p* = 0.084). Similarly, product benefit and convenience show the expected sign but are not statistically significant (H1: β = 0.132, *p* = 0.144; H3: β = 0.156, *p* = 0.135) – thus rejecting H1, H3, and H4.

The moderating effects provide some insights into these results. For completion, we first report the main effects of augmented reality and gender. The gender effect is not significant but shows the expected negative sign; thus, men are generally less likely to engage in cross-buying than women (β = -0.019, *p* = 0.777). Using a magic mirror has a statistically positive impact on consumers’ cross-buying behavior (β = 0.186, *p* = 0.029).

The empirical results show three relevant interaction effects. Gender moderates the relationship between price attractiveness and cross-buying behavior (H6b: β = -0.209, *p* = 0.036). Hence, men place less emphasis on price attractiveness when considering cross-buying options than women – confirming H6b. Surprisingly, using a magic mirror reduces the effect of product benefits on cross-buying intention (H7a: β = -0.328, *p* = 0.009). Thus, we reject H7a due to its negative effect. Consumers seem to draw more information from the interaction with a salesperson than from using a magic mirror. This is a plausible result considering previous experiences in fashion shopping. Lastly, the results show empirical support for H7c. Augmented reality positively affects consumers’ perceived convenience regarding their cross-buying behavior (H7c: β = 0.326, *p* = 0.007). Although not significant, using magic mirrors positively moderates the relationship between aesthetic quality and cross-buying behavior (H7e: β = 0.163, *p* = 0.057). Table 4 summarizes the hypotheses results.

**Table 4:** Standardized path estimates and hypotheses summary

Variable	Hypothesis	Path Estimate	*p*-value	Support	*f²*-value
Product Benefit	H1	0.132	0.169	No	0.011
Price Attractiveness	H2	0.367	< 0.001	Yes	0.102
Convenience	H3	0.155	0.158	No	0.012
Fit	H4	0.146	0.088	No	0.011
Aesthetic Quality	H5	0.169	0.010	Yes	0.033
Gender x PBF	H6a	0.159	0.211	No	0.010
Gender x PRA	H6b	-0.207	0.050	Yes	0.016
Gender x CNV	H6c	0.108	0.409	No	0.004
Gender x FIT	H6d	-0.044	0.678	No	0.001
Gender x AQ	H6e	-0.057	0.486	No	0.002
AR x PBF	H7a	-0.328	0.009	No	0.054
AR x PRA	H7b	0.024	0.789	No	0.001
AR x CNV	H7c	0.327	0.007	Yes	0.032
AR x FIT	H7d	-0.079	0.447	No	0.002
AR x AQ	H7e	0.163	0.057	No	0.018

## Discussion and implications

### Discussion

The present study validates previous results on cross-buying intention [52], indicating the importance of price attractiveness, assortment fit, and product aesthetics. Furthermore, the study disentangle moderating effects of augmented reality and gender on perceived product benefits, price attractiveness, and service convenience. Overall, this study provides a first investigation of the impact of augmented reality on consumers’ cross-buying intentions. The use of magic mirrors positively affects consumers’ cross-buying intention, but the effect is two-sided. On the one hand, magic mirrors reduce the relationship between perceived product benefits and cross-buying behavior. On the other hand, this stationary form of augmented reality increases the positive effect of perceived convenience on consumers’ buying behavior. Consequently, stationary retailers must consider whether the benefits of perceived convenience potentially outweigh magic mirrors’ negative impact. This may affect the role of magic mirrors in stationary retailing. Magic mirrors can, for example, replace sales assistants in some everyday situations or support them during peak hours.

Even though previous research indicates that marketing activities may increase perceived benefits as well as positively impact product quality [78], the use of magic mirrors reduced the relationship between product benefit and cross-buying intention, but strengthened the effect of aesthetic quality on cross-buying intention. Augmented reality does not seem to emphasize product benefits specifically. Magic mirrors may be an unobtrusive form of augmented reality but potentially fail to present additional information for the complementary product offer. In contrast, sales assistants may provide extensive feedback and resolve any additional consumer questions. Consequently, retailers have to decide which services magic mirrors can provide in combination with the sales staff. Magic mirrors may provide recommendations and additional information and may also allow customers to call for sales assistants. The sales assistant can either be available in the store or may be connected virtually as an on demand fashion advisor. In the service robot deployment model [93], an increase in human and artificial staff is targeting emotional and cognitive service encounters together.

In this study, magic mirrors demonstrate some positive impact on perceived convenience resulting in increased cross-buying intention. The set-up included correspondingly aligned products. However, retailers could experiment with whether different approaches in recommendations may be successful for the short-term shopping basket and long-term consumer loyalty.

Magic mirrors may potentially affect the perception of how well the cross-selling offer complements the original product choice. Another potential control factor for product fit could be how consumers perceive such additional service of magic mirrors: Do they provide added value [79] or are they perceived as promotional?

Irrespective of the discussion whether consumers accept digital purchase recommendations [75] or are averse to digital recommender systems [12], the direct effect of magic mirrors is positive. However, the present results potentially indicate that consumers perceive the recommendation of a salesperson as providing more product benefits than a recommendation via a magic mirror. If consumers consider recommendations by sales staff more genuine and do not perceive the recommendations as promotional, magic mirrors may only partly replace the advisory function of sales staff.

The empirical results show significant differences between women and men regarding perceived price attractiveness influencing cross-buying intention. This result in cross-buying behavior is in contrast to no gender differences in cross-buying [e.g., 72] but well in line with findings showing that women are willing to pay more for the same product when it is offered in a hedonic store atmosphere [11] and have higher expenditures on fashion purchases [63]. This gender difference may partly be explained by different shopping and technology use behaviors. For example, various consumer types perceive digitalization differently [23], and men show a higher acceptance of using a magic mirror than women [37]. The impact of female consumers, thus, seems to be more relevant, as demonstrated by the significant result regarding price attractiveness.

### Implications

Augmented reality is one potential way for stationary retailers to compete with online retailers and their ability to provide additional information online. Stationary retailers are challenged by this informational convenience of online shopping. Augmented reality, such as magic mirrors, can provide recommendations and visual fitting [79]. The use of augmented reality could, thus, help reduce the information deficits at the point of sale compared to online shopping. Additional information and services via augmented reality could further reduce consumers’ information costs. Retailers may, for example, provide information on product availability, alternative sizes, and colors – especially in combination with virtual fitting.

A primary challenge for such augmented reality technologies is how consumers perceive recommendations, for example by magic mirrors. Consumer may perceive recommendations as adding value or as promotional, intrusive efforts that lead to consumer reactance. If potential consumers perceive cross-selling offers by magic mirrors as promotional, retailers may counter this perception by empowering their customers to use augmented reality applications as they see fit. As with digital technology in general, retailers should try to create transparency and trustworthiness.

Magic mirrors particularly induce added benefits in shopping convenience by virtual try-on [33], [79], [94]. Furthermore, the present study shows how magic mirrors provide cross-selling offers and, thus, positively affect consumers’ perception of such offers and their buying intention. Retailers may utilize a variety of recommendation systems that may also include store-specific information, such as inventory level and delivery capacities.

Increased price attractiveness can potentially result in a higher price for a product or increased sales volume. The empirical results suggest that magic mirrors may realize this potential for women. In the case of artificial intelligent systems, this may, however, discriminate against gender. Additionally, this study only analyzed price attractiveness and did not study willingness to pay. Future research should analyze this interrelation further, also concerning augmented reality.

Beyond the price attractiveness, stationary retailers should pay attention to the price alignment of the original product and the cross-selling offer. Comparatively differently priced products may induce reactance in consumers. Consumers may also react similarly negatively to dynamic prices [90]. Retailers should also consider the closeness of products and product categories. Consumers may already be aware of complementary products and accessories such as those in the experimental study. Consequently, retailers need to consider how far recommendations should stretch across their assortment and whether to include in-store available or also deliverable products.

In general, retailer can use augmented reality at the point of sale to create cross-selling potential, particularly at busy times in the store. The management decision may not be one of replacing sales assistants but of a complementary role for magic mirrors. As the sales staff can only serve a limited number of consumers, retailers can combine sales staff and augmented reality technology to attend to more consumers and create cross-selling offers for each consumer. The personal resource decision may be particularly relevant for small and medium-sized businesses and highly competitive industries, such as apparel retailing. As augmented reality particularly increases shopping convenience and product recognition, retailers also have to decide which product information to provide via magic mirrors. Magic mirrors, especially in the form of virtual fitting rooms [79], could contact in-store or decentralized, on-call sales assistants that may resolve any questions. More directly, retailers can provide information capacities similar to online shops (such as availability as well as alternative colors and cuts) and integrate store-specific information, such as location in the store.

## Limitations and future research

The present study provides first insights into the impact of augmented reality on cross-buying behavior. Apart from validating the underlying cross-buying dimensions, the present results demonstrate moderating differences in cross-buying and the perception of the attractiveness of cross-selling offers when magic mirrors are present. Even though we provide a thorough exploration of cross-buying behavior in the context of magic mirrors, this study is still subject to limitations that may indicate options for further research.

Firstly, the present study considered comparatively low information content for the cross-selling offer. The magic mirrors only provided the fitting options and links to further information without including such information in the scenarios. Further research could consider a more extensive offer including various products as well as alternative cuts and colors. However, increasing informational content may also increase resistance if the information is considered intrusive or promotional in nature.

Secondly, the experimental stimuli provided a good representation of the research objectives. However, the analysis only broadly captured the effect of aesthetic product quality of the visual stimuli on the appeal of the cross-selling offer. Further research can explicitly register aesthetic perceptions and extend the studied stimuli.

Thirdly, even though the present study uses a reasonable random sample for an exploration of augmented reality applications, the sample was predominantly aged 20 to 40 years. Further research may analyze the effect of age and extend beyond a national setting. Researchers may obviously also look beyond fashion retailing, which is the prevalent field of use for magic mirrors so far.

Fourthly, the experimental scenario did not assume a prior relationship between retailer and consumer. Therefore, no factors that presuppose an existing relationship, such as consumer satisfaction and relationship length, were taken into consideration. We, therefore, call for future studies incorporating customer relationships and considering consumer characteristics beyond demographics.

Finally, we encourage future research to extend our methodological approach. For example, field experiments can better represent the retail surrounding and validate the present results. A future validation and field study can also mitigate the fear of non-response bias. We, thus, call for field research including observations and neurophysiological measurements of behavioral decision-making in retail stores.

## Electronic supplementary material

Below is the link to the electronic supplementary material.

Supplementary Material 1
