# Supplementary material for: Magic mirror on the wall: Cross-buying at the point of sale
Source: Electron Commer Res. 2023 Apr 5:1–24. Online ahead of print. doi: 10.1007/s10660-023-09687-4 (PMC10073788; doi:10.1007/s10660-023-09687-4)
Supplement: Supplementary file 1 — Supplementary Material 1 [file 10660_2023_9687_MOESM1_ESM.docx]

**EiC Comments:

I have quickly reviewed your revisions, and I think that you have made your case, though 301 responses from an online survey, I do need detailed information on all of the non-responses (indeed, you should make your data available publicly). You have not included your response to queries in the paper, so I am going to require that these be integrated along with a detailed explanation of how you handled non-responses, and how you feel this has or has not biased the results.**

**I look forward to seeing your revision.**

Thank you for your comments and your constructive feedback on the non-response bias. We included the number of responses in our original submission but removed some details in response to the first round of revision. We now included the details again. The sample section reads as follows (changes highlighted in green).

*3.3 Data Sample and Data Overview*

*A pretest suggested minor changes in question phrasing. The online survey was then carried out in April 2019. In order to ensure data quality, we collected participants' consent for the study at the beginning of the survey and directly asked about the seriousness of their participation at the end of the survey. Furthermore, the survey used items from established scales, and the items were randomly rotated in each questionnaire. We also controlled for straightlining behavior but found none in the final sample.*

*The questionnaire was opened 462 times and yielded a total of 317 completed questionnaires. All participants consented to anonymously and voluntarily participate in the study being able to terminate the study at any time without negative consequences. We removed two questionnaires based on a seriousness check and 14 minors from the sample, resulting in a final sample of 301. Participants were 52.5% (158) female and 47.5% (143) male. Following the random assignment of participants to the augmented reality and non-augmented reality scenarios, gender was also almost equally distributed in the case of the magic mirror (female 53.0% (80); male 47.0% (71)), and the sales assistant scenario without the magic mirror (female 52.0% (78); male 48.0% (72)). The majority of our participants was aged between 20 and 40 (73.1%) and resided in Germany (93.7%). We also asked for education and occupation but found no differences across these demographics. The scenarios with augmented reality and without augmented reality were randomly assigned and no significant demographic differences were detected.*

*We conducted power analysis in G*Power to determine the minimum required sample size. To explore the modeled associations at a 0.05 significance level, an effect size of 0.15, and a power level of 0.80, the suggested minimum sample size is n = 146 to perform the variance-based structural modeling analysis. The final sample of 301 questionnaires, thus, exceeds the minimum sample size of n = 146.*

We also provide the data set with this revision. The data set is also available through: https://github.com/Hattonian/MagicMirror/blob/67ab930afe276f884fb9a43615647c6ef471a965/MagicMirror_Data.csv

Regarding potential non-response bias, the senior author provides a comparison of 6 recent online surveys based on the same platform but different in topic and extent. For further comparison, we also provide 4 studies in which responses were collected from research panels.

| **Study** | **Viewed Questionnaire** | **Started Questionnaire** | **Finished Questionnaire** | **Use of a Research Panel** |
| --- | --- | --- | --- | --- |
| Study 01 | 1,740 | 809 | 530 | No |
| Study 02 | 802 | 428 | 323 | No |
| Study 03 | 829 | 330 | 248 | No |
| Study 04 | 11,662 | 6,446 | 5,130 | No |
| Study 05 | 628 | 304 | 270 | No |
| Study 06 | 838 | 426 | 233 | No |
| Study 07 | 973 | 799 | 562 | Yes |
| Study 08 | 1,023 | 820 | 596 | Yes |
| Study 09 | 973 | 799 | 562 | Yes |
| Study 10 | 2,120 | 1,811 | 714 | Yes |
| This Study | --- | 462 | 317 | No |

Table: Study Comparison

The response statistics are comparatively similar across the present study and studies 01 through 06. In contrast, participants collected from research panels show an expected smaller relative decrease from accessing the survey (“Viewed”) to starting (“Started”) the questionnaire and to completely finishing the questionnaire (“Finished”).

Additionally, we compared the empirical results from the first half of participants (n = 151) to the second half (n = 150) of participants. Please note that the usual first-third (or quarter) to last-third (or quarter) comparison is not feasible as n = 100 (or n= 75) is below the required sample size. MICOM analysis reveals at least compositional invariance based on 5,000 permutations. As for equal mean values and variances, we detect two borderline differences (mean: Fit: 0.195, p = 0.046 and variance Convenience: 0.199, p = 0.049). Due to the corresponding sample size, we assume full measurement invariance and present the results from the bootstrap multigroup analysis (5,000 subsamples) across both halves. The p-value is above 0.05 for all differences between the first and second half split. So, there is no statistical difference in answering behavior. Following some research, we can infer that no late-response bias limits the likelihood of non-response bias.

|  | **Original correlation** | **Correlation**  **permutation mean** | **5.0%** | **Permutation p-value** |
| --- | --- | --- | --- | --- |
| AR | 1.000 | 1.000 | 1.000 | 0.272 |
| Convenience | 0.999 | 1.000 | 0.999 | 0.191 |
| Cross Buying | 1.000 | 1.000 | 1.000 | 0.944 |
| Fit | 1.000 | 0.999 | 0.997 | 0.996 |
| AQ | 1.000 | 0.997 | 0.990 | 0.866 |
| Gender | 1.000 | 1.000 | 1.000 | 0.150 |
| Price Attractiveness | 1.000 | 1.000 | 1.000 | 0.419 |
| Product Benefit | 1.000 | 1.000 | 0.999 | 0.439 |

Table: MICOM Step 2

|  | **Original difference** | **Permutation mean**  **difference** | **5.0%** | **95.0%** | **Permutation p-value** |
| --- | --- | --- | --- | --- | --- |
| AR | -0.003 | 0.000 | -0.096 | 0.090 | 0.521 |
| Convenience | 0.002 | -0.000 | -0.193 | 0.191 | 0.490 |
| Cross Buying | -0.080 | -0.001 | -0.191 | 0.192 | 0.245 |
| Fit | 0.195 | -0.001 | -0.188 | 0.190 | 0.046 |
| AQ | 0.011 | -0.001 | -0.187 | 0.190 | 0.460 |
| Gender | 0.023 | -0.000 | -0.097 | 0.090 | 0.386 |
| Price Attractiveness | 0.175 | -0.001 | -0.187 | 0.192 | 0.067 |
| Product Benefit | 0.026 | 0.000 | -0.189 | 0.190 | 0.408 |

Table: MICOM Step 3a (Mean)

|  | **Original difference** | **Permutation mean**  **difference** | **5.0%** | **95.0%** | **Permutation p-value** |
| --- | --- | --- | --- | --- | --- |
| AR | 0.000 | -0.000 | -0.001 | 0.001 | 0.473 |
| Convenience | 0.199 | -0.000 | -0.200 | 0.198 | 0.049 |
| Cross Buying | 0.168 | -0.002 | -0.173 | 0.176 | 0.058 |
| Fit | -0.002 | 0.000 | -0.229 | 0.234 | 0.491 |
| AQ | 0.188 | 0.002 | -0.225 | 0.231 | 0.089 |
| Gender | 0.005 | -0.000 | -0.020 | 0.018 | 0.386 |
| Price Attractiveness | 0.086 | -0.003 | -0.217 | 0.204 | 0.242 |
| Product Benefit | 0.098 | -0.000 | -0.246 | 0.239 | 0.259 |

Table: MICOM Step 3b (Variance)

|  | **Difference**  **(First_Half - Last_Half)** | **2-tailed**  **(First_Half vs Last_Half)**  **p-value** |
| --- | --- | --- |
| AR -> Cross Buying | -0.022 | 0.897 |
| Convenience -> Cross Buying | 0.215 | 0.374 |
| Fit -> Cross Buying | 0.075 | 0.694 |
| AQ -> Cross Buying | -0.096 | 0.478 |
| Gender -> Cross Buying | -0.268 | 0.053 |
| Price Attractiveness -> Cross Buying | -0.121 | 0.526 |
| Product Benefit -> Cross Buying | -0.046 | 0.809 |
| AR x Price Attractiveness -> Cross Buying | 0.319 | 0.133 |
| Gender x Convenience -> Cross Buying | -0.266 | 0.340 |
| Gender x Product Benefit -> Cross Buying | 0.347 | 0.216 |
| AR x Fit -> Cross Buying | -0.069 | 0.760 |
| Gender x Fit -> Cross Buying | -0.167 | 0.472 |
| AR x AQ -> Cross Buying | 0.227 | 0.198 |
| Gender x AQ-> Cross Buying | 0.196 | 0.209 |
| Gender x Price Attractiveness -> Cross Buying | -0.140 | 0.539 |
| AR x Convenience -> Cross Buying | -0.316 | 0.233 |
| AR x Product Benefit -> Cross Buying | -0.096 | 0.714 |

Table: Bootstrap Multigroup Analysis

We added a brief statement to our Limitations and Future Research section which now reads as follows.

*Finally, we encourage future research to extend our methodological approach. For example, field experiments can better represent the retail surrounding and validate the present results. A future validation and field study can also mitigate the fear of non-response bias. We, thus, call for field research including observations and neurophysiological measurements of behavioral decision-making in retail stores.*

**AE Comments:**

**Thank you for submitting another revised version of your paper to the ECR. We acknowledge the improvements made to the manuscript and the detailed response to both the reviewers’ and editors’ queries.**

**Accordingly, we are glad to recommend the acceptance of your manuscript. As the AE for this submission, we wish to congratulate the authors for this great achievement. Well done!**

We appreciate the constructive process and comments of the editor and the reviewers! Thank you very much for your support and for helping to improve our manuscript.
